# The complete plastid genome of *Ruppia brevipedunculata* Shuo Yu & den Hartog 

**DOI:** 10.1080/23802359.2019.1704653

**Published:** 2020-01-10

**Authors:** Shuo Yu, Xiaojuan Li, Kai Jiang, Miaomiao Shi

**Affiliations:** a Ministry of Natural Resources, Fourth Institute of Oceanography, Beihai, China;; b Key Laboratory of Plant Resources Conservation and Sustainable Utilization, South China Botanical Garden, Chinese Academy of Sciences, Guangzhou China;; c College of Life Sciences, University of Chinese Academy of Sciences, Beijing, China;; d Shanghai Chenshan Plant Science Research Center, Chinese Academy of Sciences, Chenshan Botanical Garden, Shanghai, China;; e School of Ecological and Environmental Sciences, Shanghai Key Lab of Urban Ecological Processes and Eco-Restoration, East China Normal University, Shanghai, China;; f Center of Conservation Biology, Core Botanical Gardens, Chinese Academy of Sciences, Guangzhou, China

**Keywords:** *Ruppia brevipedunculata;* Illumina sequencing, plastid genome, phylogenetic relationship

## Abstract

The taxonomy of *Ruppia* has long been confused due to its high plasticity in morphology. In this study, the complete plastid genome sequence of *Ruppia brevipedunculata* was successfully sequenced by the technology of Illumina. The whole plastid genome length was 158,943 bp and contained a typical quadripartite structure including one large single copy (LSC) region (88,857 bp), one small single copy (SSC) region (19,130bp) and a pair of inverted repeats (IR) regions (25,478bp). The GC content of this genome was 35.8%. The whole genome contained 132 genes including 87 protein-coding genes, 37 tRNA genes, and 8 rRNA genes. The phylogenetic analysis indicated that *R. brevipedunculata* and *R. sinensis* formed a distinct clade separated from *Potamogeton perfoliatus*.

*Ruppia* is considered as the most widely distributed seagrass, which usually grows in coastal lagoons of tropical and temperate zones (Short et al. 2007). *Ruppia* species can co-occur with other seagrasses such as *Zostera marina* and *Halophila ovalis* in the shallow sea waters. The *Ruppia*-dominated ecosystems support high biodiversity by providing habitat or food for fish, invertebrates and migratory birds (Triest et al. 2018). So far, the taxonomy of this genus has long been confused due to the high intraspecific phenotypic plasticity and interspecific hybridization (Ito et al. 2010; Triest and Sierens 2010; Yu and den Hartog 2014). In this study, we sequenced the complete plastid genome of *R. brevipedunculata* using next-generation technology. This genome will be very useful in taxonomy and phylogenetic studies for *Ruppia*.

Fresh plant of *R. brevipedunculata* was collected in an abandoned saltern from Dongfang, Hainan Province, China (18.94°N, 108.65°E), and the voucher specimen was stored at Fourth Institute of Oceanography Herbarium (DF201908-1). After cleaning the attached epiphytes, leaves were washed with fresh water. Then, the genomic DNA was extracted according to a modified CTAB method and sequenced using the Illumina Novaseq platform. Low-quality reads and adapters were trimmed off by the FastQC software (Andrews 2010). *De novo* genome assembly was conducted by SPAdes v3.9 (Bankevich et al. 2012). The complete plastid genome was annotated with DOGMA (Wyman et al. 2004). The annotations of tRNA genes were performed by ARAGORN (Laslett and Canback 2004). The complete plastid genome of *R. brevipedunculata* was submitted to GenBank database (Accession Number: MN736637).

The complete plastid genome of *R. brevipedunculata* was 158,943 bp in length with a typical structure including one large single-copy region (88,857 bp), one small single-copy region (19,130 bp) and a pair of inverted repeats (IRs) (25,478 bp). The guanine-cytosine (GC)-content was 35.8%. There was a total of 132 genes in this genome consisting of 87 protein-coding genes, 37 tRNA genes, and 8 rRNA genes. There were 18 duplicated genes in the IR regions including seven protein-coding genes (rpl2, rpl23, ycf2, ycf15, ndhB, rps7 and rps12), seven tRNA genes (trnI-CAU, trnL-CAA, trnV-GAC, trnI-GAU, trnA-UGC, trnR-ACG and trnN-GUU), and four rRNA genes (rrn16, rrn23, rrn4.5 and rrn5).

To clarify the phylogenetic position of *R. brevipedunculata*, we then downloaded 22 completed plastid genomes from GenBank database. The phylogenetic tree was reconstructed with RAxML software (Stamatakis 2014) using maximum-likelihood (ML) method. Bootstrap values were calculated using 1000 replicates. As shown in the phylogenetic tree (Figure 1), *R. brevipedunculata* and* R. sinensis* formed a distinct clade separated from *Potamogeton perfoliatus*.

**Figure 1.**
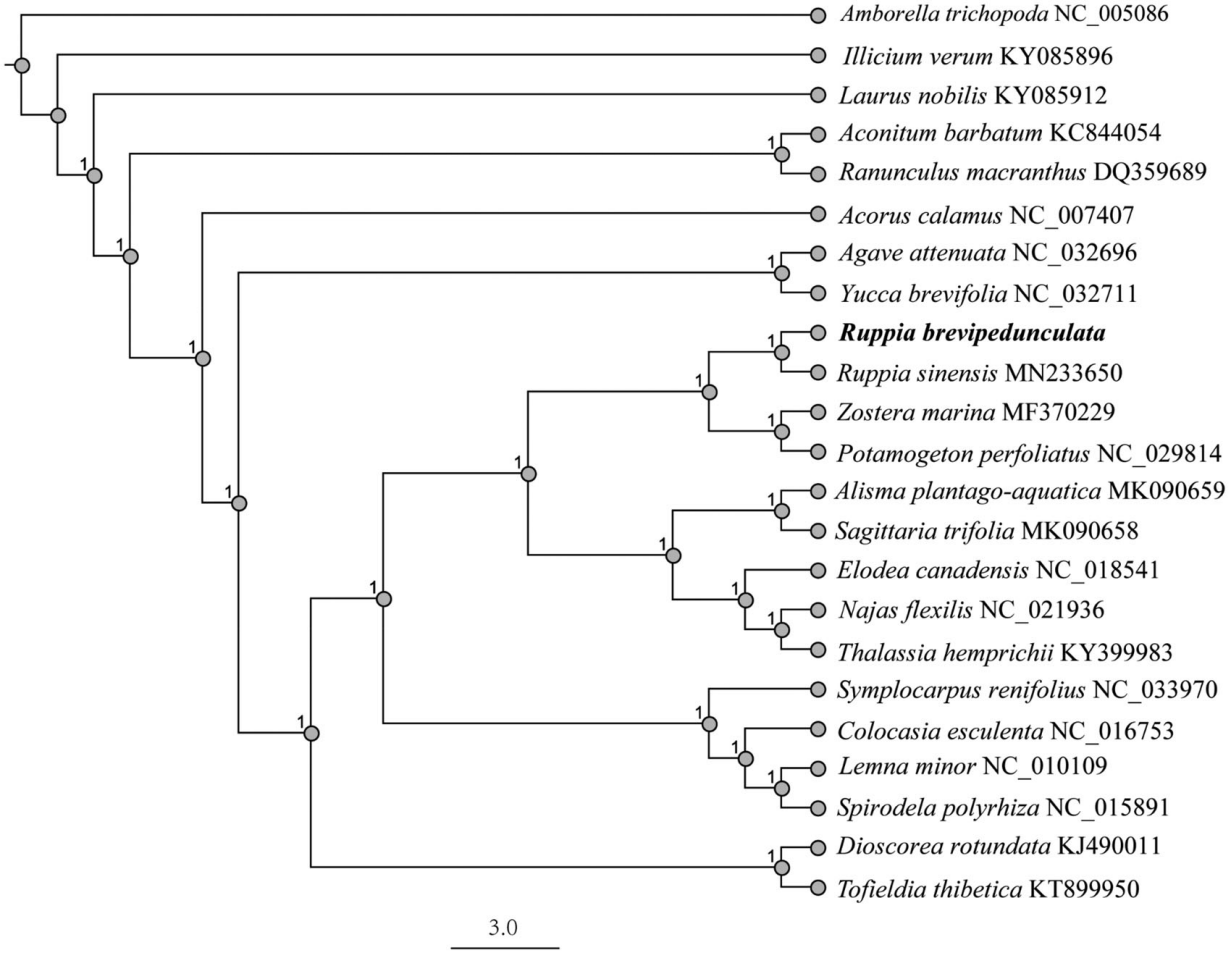
Phylogenetic relationship of 23 species based on the plastid genome sequences with maximum-likelihood (ML) analysis.

## Geolocation information

Dongfang, Hainan Province, China (18.94°N, 108.65°E)
